# Space-Time Modelling of the Spread of Salmon Lice between and within Norwegian Marine Salmon Farms

**DOI:** 10.1371/journal.pone.0064039

**Published:** 2013-05-20

**Authors:** Magne Aldrin, Bård Storvik, Anja Bråthen Kristoffersen, Peder Andreas Jansen

**Affiliations:** 1 Norwegian Computing Center, Oslo, Norway; 2 Department of Mathematics, University of Oslo, Oslo, Norway; 3 Norwegian Veterinary Institute, Oslo, Norway; 4 Department of Informatics, University of Oslo, Oslo, Norway; University of Toronto, Canada

## Abstract

Parasitic salmon lice are potentially harmful to salmonid hosts and farm produced lice pose a threat to wild salmonids. To control salmon lice infections in Norwegian salmonid farming, numbers of lice are regularly counted and lice abundance is reported from all salmonid farms every month. We have developed a stochastic space-time model where monthly lice abundance is modelled simultaneously for all farms. The set of farms is regarded as a network where the degree of contact between farms depends on their seaway distance. The expected lice abundance at each farm is modelled as a function of i) lice abundance in previous months at the same farm, ii) at neighbourhood farms, and iii) other, unspecified sources. In addition, the model includes explanatory variables such as seawater temperature and farm-numbers of fish. The model gives insight into factors that affect salmon lice abundance and contributing sources of infection. New findings in this study were that 66% of the expected salmon lice abundance was attributed to infection within farms, 28% was attributed to infection from neighbourhood farms and 6% to non-specified sources of infection. Furthermore, we present the relative risk of infection between neighbourhood farms as a function of seaway distance, which can be viewed as a between farm transmission kernel for salmon lice. The present modelling framework lays the foundation for development of future scenario simulation tools for examining the spread and abundance of salmon lice on farmed salmonids under different control regimes.

## Introduction

Infectious diseases constitute a constant problem in industrialised farming where there typically are both high densities of farms and high densities of animals within each farm. Disease outbreaks can have large economic consequences to farming industries, but can also have severe ecological effects if infections are spread to and impair the viability of wild animals. Several mathematical and statistical models within this field have been developed during the last decades and applied to as diverse diseases as foot-and-mouth disease, swine fever, bluetongue and infectious salmon anaemia [1]–[5]. In all these models, probabilities of infection relative to distance, often called transmission kernels [1], [4], play an important role.

Salmon lice (*Lepeophtheirus salmonis*) are parasitic copepods that live on the skin surface of both wild and farmed salmonids. The parasite uses rasping mouthparts to feed on mucus, skin and underlying tissues of its host and thereby causes mechanical damage [6]. Possible effects of salmon farming on sea lice infections on wild stocks of salmonids, and hence the viability of such wild stocks, has evoked a large and contentious debate [7]–[9]. Nevertheless, the notion that salmon farming does affect local transmission of salmon lice to wild salmonids [9], [10], as well as to farmed salmonids [11], seems well established. Recent studies also report that parasiticide-treatment of outwardly migrating salmon smolts significantly increases their marine survival compared to non-treated control smolts, suggesting that salmon lice induce mortality in wild salmonid hosts [12]–[14]. Due to the potential impact of salmon lice of farm origin on wild stocks of salmonids, salmon lice infections on farmed fish are strictly regulated in Norway [11]. To enforce these regulations, numbers of salmon lice are counted on samples of farmed salmonids at regular intervals from all actively producing marine fish farms each month. Salmon lice abundances from these counts, i.e. the average number of salmon lice per fish, are reported to a central data base [11]. Measures to control salmon lice infections, i.e. the application of medical treatments or the use of cleaner-fish to prey on lice, are also reported to this same data base.

The large spatio-temporal dataset covering salmon lice abundance on salmon farms, and efforts to control these infections, should give insight into factors that affect farm levels of salmon lice infections and the contributing sources for such infection. The aim of the present study was to develop a modelling framework that could: i) disentangle different contributing sources of salmon lice infections; ii) estimate functional relationships between expected salmon lice infections and contributing factors, e.g. the between farm transmission kernel as a function of seaway distance between farms [1], [4]; and iii) lay the foundation for a scenario simulation tool to examine the potential spread of salmon lice within and between salmon farms to assess the impact of control measures. We developed a stochastic space-time model where the monthly salmon lice abundances at all Norwegian salmonid farms are modelled simultaneously. The model is similar in flavour to previous models developed by the same group of authors on infectious salmon anaemia and other salmonid diseases where farms appear as infected or non-infected [5], [15], [16]. Salmon lice, however, differ from these other diseases in that most farms are infected more or less all the time, but salmon lice abundances vary both over time and between farms.

The set of salmonid farms is regarded as a network where the degree of contact between each pair of farms depend on the seaway distance between them. The observed number of lice in a given sample of fish is assumed to be distributed according to a zero-inflated negative binomial distribution. The expected salmon lice abundance at each farm is modelled as a function of i) the observed lice abundances in previous months at the same farm, ii) the observed lice abundances in previous months at neighbouring farms, and iii) other, unspecified sources of infection. In addition, the model includes explanatory variables such as seawater temperature and numbers of fish at each farm. The model was fitted to data covering all marine salmonid farms in Norway from June 2003 until the end of 2011.

In the following sections, we first describe the data and then the model. Then, we present the fitted model and other results, and finally we draw some conclusions.

## Materials and Methods

### Salmonid Farming and the Problem of Salmon Lice

During a 30 year period from 1980 to 2010, annual production of farmed salmonids, i.e. Atlantic salmon (*Salmo salar*) and rainbow trout (*Onchorhyncus mykiss*), has grown from marginal to around one million tonnes in Norway [17]. For comparison, the total annual yield of wild Atlantic salmon and sea trout (*Salmo trutta*) in Norway was estimated at 850–1400 tonnes between 2000 and 2008 [18]. The production of salmonids consists of a freshwater juvenile phase, followed by a marine grow out phase, which is the focus of this study. Marine salmonid farming is regulated through a system of legal concessions authorised by the Norwegian Directorate for Fisheries (DFF; http://www.fiskeridir.no). All farms are registered with a geographic reference in the Aquaculture register, which is available at DFF's website. For marine farms that actively produce salmonids, it is mandatory to report key statistics on their fish stocks, fish health related statistics and seawater temperature at 3 m depth, on a monthly basis.

The production of a salmonid cohort on a farm typically initiates by stocking juvenile smolts to net-pens on the farm in spring or in the autumn. The net-pens allow free water exchange with the surroundings. After stocking, the fishes are on-grown on the farm for roughly 1.5 years and then slaughtered. The production time varies slightly according to the weight of the fish at slaughter, seawater temperature etcetera. On a given farm, only fish of a given year class are produced. After slaughtering, it is mandatory to fallow the farm location for a short period before stocking a new cohort of salmonids. Occasionally fish may be moved from one farm location to another, in which case a new farm will initially report fish weights larger than juveniles.

Sea lice are naturally occurring parasitic copepods that spread by planktonic larvae. Farmed salmonids are infected by water flow through the net-pens. The parasites live on the surface of the fish and feed on mucus, skin and underlying tissues of its host [6]. Sea lice infections on farmed salmonids are dominated by the salmonid specific salmon louse, but may also be by the generalist parasite *Caligus elongatus*. However, regulations on reporting requirements regarding sea lice infections on farmed salmonids in Norway (see below) are only directed at the salmon louse. Hence, we term the parasitic infections for salmon lice in this paper. The salmon louse has a life cycle consisting of three planktonic larval stages, of which the third copepodite-stage may attach to the surface skin of a salmonid host by a frontal filament. Upon infection, the parasite develops through four consecutive stages of attached larvae, followed by two pre-adult stages of lice that may move about on the surface of the fish (mobile stages), and finally adult males and females that also are mobile. Sea lice infections on farmed salmonids are regulated through a system of maximum thresholds of abundance of mobile stages. To enforce these regulations, farmers are instructed to count salmon lice on farmed salmonids at regular intervals. Counts are reported once a month to a database as the mean number of lice per fish within two stage categories: i) adult females, and ii) all other mobile stages (pre-adults and adult males). The farmers are instructed to report the highest mean count of adult females during a given month. In the present model, we sum the two categories of salmon lice and term this salmon lice abundance. The mean count of salmon lice from a sample of 20 fish from one net pen (before August 2009) or the mean of samples of multiples of 10 fish from half of the net-pens (from August 2009) was reported. Details of the sampling, counting and reporting procedures for salmon lice infections on farmed fish are given in Jansen et al. ([11]; and the Electronic supplementary material therein).

### Data

The present data include all 1401 Norwegian marine fish farms with standing stocks of either Atlantic salmon or rainbow trout (salmonids) in any month from February 2003 to December 2011. The data is an updated version of the data presented in Jansen et al. [11],who analysed data up to 2010 in a study based on a different modelling approach than the present.

**Figure 1:**
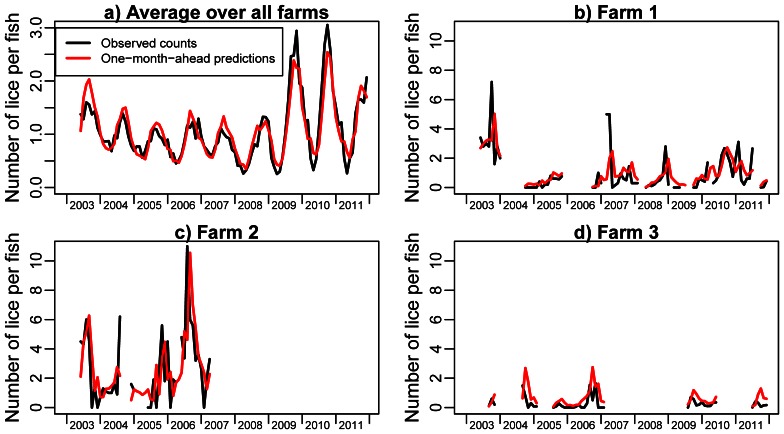
Time plots of observed (black) and one-month-ahead predictions (red) of salmon lice abundance averaged over all farms (panel a), and for three selected farms (panels b, c and d).

The data from the first four months were only used to construct lagged explanatory variables, whereas the model was fitted to data from June 2003 and onwards. In the following description, we therefore only summarise data from the latter period. The number of active farms was then reduced to 1361. Each salmonid farm normally had several consecutive periods of production of fish populations, interrupted by periods of fallowing (no fish on the farm). The fish population within a production period is termed a cohort and the present data consists of 4 255 cohorts with a total of 58 623 farm-months of cohort production. Farms produced between one and nine consecutive cohorts. For each salmonid farm, the salmon lice abundance on a sample of fish is obligatory to report to the responsible Norwegian Food Safety Authority. We assumed that the number of sampled fish always was 20. The reported salmon lice abundances were therefore multiplied by 20 and implemented as the response variable in the present model. Hence, we ignored that the number of fish sampled from August 2009 could be both less and more than 20 (see previous section), but note that this did not affect the mean number of lice per fish. However, lice abundance data were missing for about 3% of the total number of months with farmed fish, giving 56 836 farm-months with reported salmon lice abundance. Salmon lice abundances amount to between 0 and 7 in 95% of the farm-months, with a mean of 1.1 lice per fish. The distribution of salmon lice abundance is profoundly skewed, being exactly 0 in 32% of the farm-months, whereas the highest reported abundance is 163 lice per fish. The black curves in Figure 1 show how the observed lice abundance vary over the data period averaged over all farms and for three selected farms. The lice counts have a clear seasonal pattern driven by the seawater temperature. The farm-wise panels also show how the fish cohorts can be distributed over the data period. Farms 1 and 2 in the figure have periods where the farms are active, but the salmon lice counts are missing. This is shown by predictions (red line) but missing observations (no black line) in the time plots.

**Figure 2:**
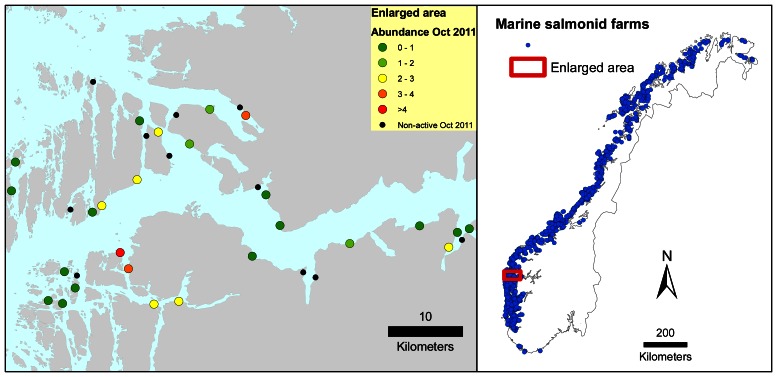
Location of salmonid farms. The left panel shows marine farms in the enlarged Sognefjorden area that were actively producing salmonids between June 2003 and December 2011, with observed salmon lice abundance indicated for those active in October 2011. The right panel shows marine farms that were active in the period June 2003 - December 2011 in the whole of Norway.

The geographic location of each farm and pairwise seaway distances between all farms were compiled from Jansen et al. [11]. The seaway distance between two farms is defined as the shortest distance through water. The various salmonid farms had between zero and 31 other salmonid farms within a seaway distance of 10 km (median five). Figure 2 shows the locations of all Norwegian salmonid farms that were actively producing salmonids at some month during the study period, with a closer look at the 37 farms that were active in the Sognefjorden area. Of the latter, 19 farms were actively producing in October 2011.

Monthly figures of several other variables were also given separately for each farm. Each farm could produce either Atlantic salmon or rainbow trout, or both species at the same time. We calculated the monthly proportion of Atlantic salmon biomass for each farm, which was 91% on average. The monthly average weight of fish was between 100 g and 5.8 kg for 95% of the farm-months, with a mean of 2.0 kg. The mean number of fish per farm was 530 000 (95% between 200 000 and 1.6 million). The mean seawater temperature at farms was 9.1°C (95% of all temperatures was between 3.4°C and 16.3°C). The seawater temperature was missing for 2.3% of the farm-months. Each missing temperature was imputed by a weighted mean of all observed temperatures the same month, with weights proportional to the inverse of the seaway distance to the current farm with the missing temperature. For about 4% of the farm-months, there were no fish at the same farm in the previous month in combination with the mean fish weight being less than 250 g. This indicates that the fish cohort was stocked for the first time on the given farm. Furthermore, for about 2% of the farm-months, there were no fish at the same farm in the previous month, but the mean fish weight was equal to or larger than 250 g. This indicates that the fish cohort had been relocated, i.e. moved from another marine farm. Medical salmon lice treatments were applied in 15% of the farm-months. Cleaner fish of the family *Labridae* were also applied at several farms [11], but we did not have sufficiently reliable data on the use of these, and we therefore ignored the use of cleaner fish in our modelling. Nor did we have sufficient data on salinity, which is known to affect the infection process of salmon lice [19].

### Overview of the Model

Here we first describe the main features of the present salmon lice model, and then go into more detail. The model includes all actively producing Norwegian marine salmonid farms simultaneously, but the counted number of salmon lice at a given farm in a given month is modelled conditionally upon the situation at the current farm of interest and all other farms in previous months.

Now, consider farm *i* at time or month *t*, and let 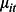 denote the expected abundance of salmon lice. This expectation is modelled as a function of i) observed lice abundances in previous months at the current farm, ii) observed lice abundances in previous months at neighbouring farms, and iii) other factors such as seaway distances to neighbouring farms, seawater temperatures etcetera.

The response variable is constructed from the reported salmon lice abundances by multiplying it by the number of fish sampled, which is always assumed to be 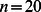, see previous section and Jansen et al. [11]. The response variable is denoted by 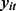, and modelled as a zero-inflated, negative binomially distributed variable [20] with expectation 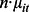. This means that the values of 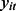 tend to be zero more often than can be modelled by a negative binomial distribution alone. Let 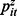 denote the probability of excess zero observations in this compound distribution. These excess zeroes come in addition to those expected from the negative binomial part of the distribution. Thus, 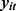 comes from a negative binomial distribution with probability 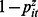, and has an excess zero value with probability 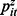. Let further 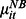 denote the expectation in the negative binomial part of the distribution. Then, the expectation of 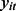 can be expressed as
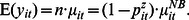
(1)


Our main focus is on modelling the expected salmon lice abundance 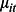, and we give a detailed description of the model for 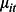 in the next subsection. Furthermore, the probability of excess zero observations is modelled as a function of 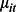 and a few other factors. Then, according to Eq. (1), the expectation in the negative binomial part of the compound distribution is given as 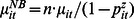. The negative binomial distribution has one parameter in addition to the expectation, here called 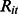, and this is also modelled a function of 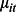. We present the sub-models for 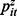 and 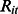 in Appendix S1, since these only affect the shape of the distribution of 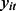, which is not our focus here. The parameter *R* has often been denoted *k* in parasitological literature [21], [22].

The motivation for using a negative binomial distribution begins with assuming that the number of lice counted on one fish, conditioned on the expected lice abundance, is Poisson distributed. However, we expect large variability from fish to fish, where typically some fish carry few salmon lice and some fish carry many. The negative binomial distribution is well suited to model such over-dispersed Poisson counts. Finally, if we sum the individual counts over 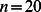 fish, the total number of counted lice is also negative binomially distributed. However, when analysing the data, we found that there was an excess frequency of zeroes, which lead us to the zero-inflated, negative binomial distribution.

The zero-inflated, negative binomial distribution was also used by Jansen et al. [11], who modelled the same data as the present, although only updated up to December 2010. However, several other aspects of their approach differ from ours; i) they did not model all farms simultaneously in a network, ii) they ignored the lice counts at neighbouring farms, and iii) they had one model for the excess zeroes and another for the expectation in the negative binomial distribution, yielding a complex formula for the expected lice abundance, whereas we model the latter explicitly.

### The Expected Lice Abundance

The model for the expected lice abundance at farm *i* in month *t* has the following additive-multiplicative form:
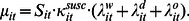
(2)


The two multiplicative terms in Eq. (2) are:

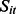
 is an “at-risk” indicator that is 1 when farm *i* is active (has a positive number of farmed fish) at month *t* and 0 otherwise.

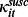
 is a factor proportional to the susceptibility of farm *i*. It depends on explanatory variables that characterise the conditions for the fish at farm *i* at month *i*. Some explanatory variables are common for all farms (e.g. season), whereas others are farm-specific (e.g. seawater temperature). The farm specific term has the form
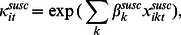
(3)where 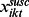 denotes explanatory variables for farm 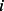 at month 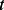 and 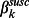 denotes corresponding regression coefficients. See Table 1 for a list of variables included in the final model.

**Table 1:** Estimated parameters in the expected abundance 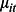 with 95% confidence intervals for the selected model, with corresponding relative BIC values for selected parameters.

							Relative BIC if deleted	
Parameter	Variable name or	Farm specific	Parameter					
group	parameter description	variable	symbol	Est.	Lower	Upper		
misc.	Other sources		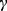	0.076	0.068	0.084		
-''-	Lagged lice counts		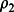	0.076	0.062	0.090		
-''-	-''-		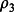	0.025	0.016	0.034		
-''-	-''-		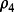	0.022	0.013	0.031	39	
-''-	Non-linear dependency		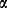	0.650	0.636	0.665		
-''-	Sea distance function		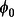	−1.444	−1.605	−1.283		
-''-	-''-		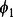	−0.351	−0.272	−0.430		
-''-	-''-		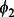	0.568	0.478	0.658		
susc.	intercept	no	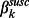	−0.385	−0.439	−0.332		
-''-	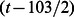	-''-	-''-	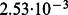	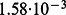	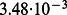		
-''-	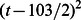	-''-	-''-	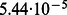	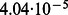	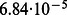		
-''-	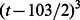	-''-	-''-	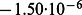	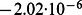	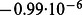	22	
-''-	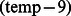	yes	-''-	0.0979	0.0937	0.1020		
-''-	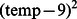	-''-	-''-	−0.0047	−0.0056	−0.0038	91	
-''-	(latitude-64)	-''-	-''-	0.0084	0.0042	0.0127	4	
-''-	(temp-9)×(latitude-64)	-''-	-''-	0.0124	0.0112	0.0135	480	
-''-	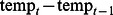	-''-	-''-	−0.0220	−0.0278	−0.0162	45	
-''-	log(weight)	-''-	-''-	0.215	0.201	0.228	967	
-''-	Stocked	-''-	-''-	−0.978	−1.115	−0.841	188	
-''-	Relocated	-''-	-''-	0.218	0.101	0.336	4	
-''-	Salmon proportion	-''-	-''-	0.130	0.083	0.176	20	
inf.	log(number of fish)	yes	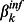	0.258	0.191	0.326	57	

misc.: Miscellaneous parameters.

susc.: Parameters related to the susceptible farm.

inf. : Parameters related to the infectious farm.

Est.: Estimate.

Lower: Lower bound of 95% confidence interval.

Lower: Upper bound of 95% confidence interval.

The three additive terms represent three possible sources of lice infection:

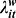
 represents infection within the current farm of interest.

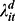
 represents infection from neighbouring farms, depending on among others the seaway distances to these farms and on their lice abundances.

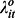
 represents infection from other, non-specified sources, for instance a reservoir of infection on free roaming salmonids.

We assume that the infection pressure from each source can be added. Details on how each of these terms are modelled are given in the following.

Infection within a farm is modelled as
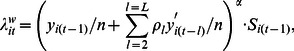
(4)where

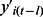
 is equal to 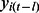 if farm 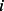 has been active in all the months from 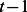 to 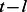, but is zero if the farm has been in-active in any of these months, and

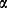
 is a positive parameter that allows for a non-linear dependency of the previous months' lice counts.

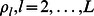
 are parameters that account for the effect of previous lice abundances in sequential time steps. The term 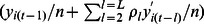 divided by 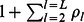 is a weighted sum of the lagged observed lice abundances. Allowing for more than one lag may be useful, since the observed lice abundance is based on counts on small samples of fish, hence using several months may reduce the sampling uncertainty.

**Figure 3:**
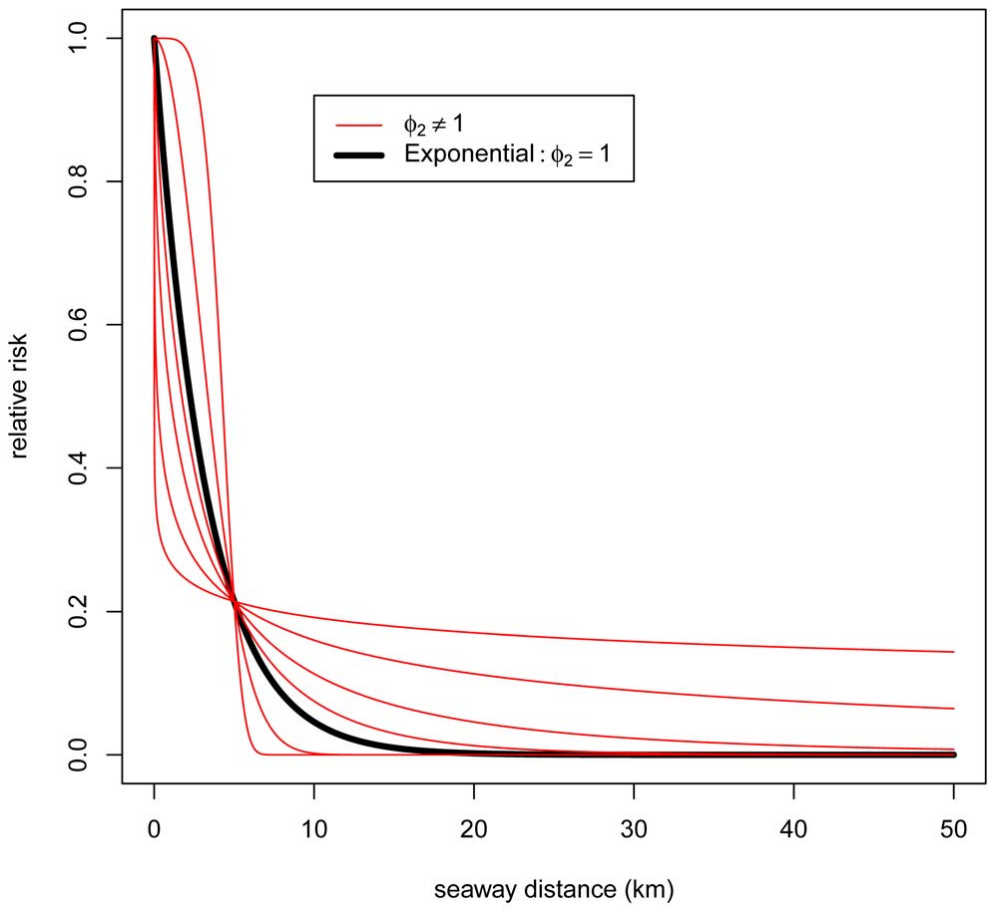
A selection of possible shapes for the relative effect of the seaway distance for various values of the 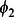 parameter.

The term representing *infection from neighbouring farms* is summed over the separate contribution from all other farms and modelled as
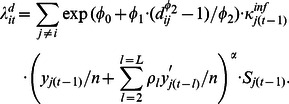
(5)Here,

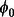
 quantifies the importance of neighbouring infection compared to the other two sources of infection.

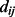
 is the seaway distance between farms 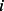 and 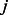.

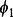
 and 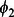 are parameters that reflect the effect of the seaway distances to the neighbouring farms. The transformation 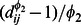 is the well known Box-Cox transformation, which allows for many different shapes of the distance function (Figure 3). When 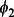 approaches zero, the Box-Cox transformation becomes 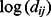, where 1og here and elsewhere means the natural logarithm, and the distance function then becomes proportional to 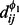. When 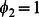, the distance function becomes proportional to the exponential function 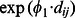.

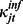
 is a factor proportional to the infectiousness of farm *j*, depending on explanatory variables that characterise the neighbouring farm *j*, for instance the number of fish. The infectiousness term has the form
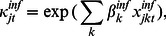
(6)where, as before, 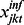 denote explanatory variables and 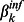 denote corresponding regression coefficients. See Table 1 for an overview of variables included in the final model and the Results section for other variants of the model that were investigated.

*Infection from other sources*, 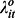, is currently modelled as a constant 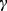, and as such acts as an intercept term. This can, however, be modified to a more complex form, for instance by including functions of space and time.

### Relative Contribution from Each Source

For each farm and each month, the expected lice abundance can be decomposed into the relative contribution from each of the three infection sources. Summing over all farms and months gives the mean relative contribution from each source. For instance,
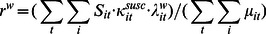
(7)is the mean relative contribution within a farm. The mean relative contribution from neighbouring farms, 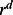, and from other sources, 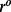, are defined similarly.

### Estimation

The unknown parameters consist of 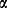, 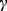, 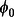, 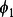, 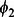, 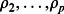 and all 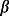-s, joined into a parameter vector 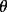. The maximum likelihood estimates of 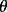 was found by maximising the log likelihood of the data. An expression of the log likelihood is given in Appendix S1.

The log likelihood was maximised using the function *optim* in the statistical software *R*, using the method of Byrd et al. [23] for optimisation. Parameter uncertainties were based on the observed information matrix [24], and are reported as 95% Wald confidence intervals.

We investigated several variants of this model, with different explanatory variables included and with some parameters set to 0. For model selection, we used the Bayesian information criterion (BIC) [25], [26]. This criterion balances the fit of the data to the number of parameters by minimising 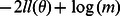, where 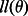 is the log likelihood and *m* is the total number of observations and *q* the number of parameters. For some important explanatory variables, we considered second and third order terms as well as cross products between pairs of variables. We followed a strategy where main effects were included first, and second higher order effects or cross products were included if this improved the BIC value. Explanatory variables that were included in second or third order terms or cross products were centred to reduce correlations between the variables. We could alternatively have used Akaike's Information Criterion (AIC) [26], [27] for model selection, which penalises the number of parameters less than BIC and therefore tends to give models with more parameters than when BIC is used. Choosing between BIC and AIC is primarily a matter of taste. We chose BIC because our dataset was large and hence we expected to end up with a rather complex optimal model also by using BIC.

Medical lice treatment was applied in 15% of the farm-months, which in principle should lead to reduced lice counts after treatments. It would indeed be useful if the model could be used to quantify the effect of such treatments. However, there is a dual relationship between treatment and lice counts; a high lice count will often induce a following treatment, whereas a treatment may result in lower lice counts. We don't know the actual dates for neither lice counts nor treatments, so it is not known for certain whether a treatment was applied before or after the corresponding lice count. Furthermore, we do not know what type of chemotherapeutic treatment that is used in each case. This makes it difficult to interpret the estimated effect of medical treatment. Therefore, we first consider a model without medical treatment as a factor, but then return to a model that includes medical treatment later on.

We estimated the model by fitting it to the observed lice counts from the 103 months between July 2003 and December 2011. We investigated models with up to 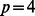 lags of previous lice counts, so the four months of data from February to June 2003 were only used to construct these lagged lice counts. As mentioned in the Data Section, 3% of the lice counts were missing. It is reasonable to believe that these were missing at random in the context of Little and Rubin [28], and the corresponding farm-months were therefore ignored in the likelihood. However, for the lagged lice counts, each missing value was imputed by the last observed value within the same cohort, if any. If this was impossible, the missing value was imputed by the mean of all observed lice counts in the current month.

## Results

We first consider a model without medical treatment. Then, we investigated several variants of the model and selected the model with the minimum BIC value. Figure 1 shows time plots of the estimated expected number of lice per fish and the corresponding observed lice abundance averaged over all farms and for three selected farms. In our model, the expected numbers of lice per fish are the same as one-month-ahead predictions, and there is therefore a tendency for the expected values being shifted to the right compared to the observations. The selected model is given in Table 1, with estimates and 95% confidence intervals for each coefficient that enters the model for the expected salmon lice abundance. Estimates for eight additional coefficients in the sub-models for the excess zero probability and 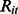 are given in Table A in Appendix S1. For some coefficients, the table also shows how much the BIC value would increase if this coefficient was excluded (set to 0) from the model, here called the relative (to the main model) BIC values. A high value of the relative BIC indicates that the corresponding variable improves the model fit significantly. Some effects that are handled by groups of explanatory variables, for instance the seawater temperature effect, and the relative BIC values for such effects are presented in the text below. The confidence intervals are rather tight, and all parameters are highly significantly different from natural reference values of 0 or 1 (the latter is relevant for the 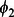 parameter in the Box-Cox transformations, where a value of 1 implies linearity on the exponential scale, i.e. an exponential distance function). We have therefore not included any p values in the table.

In the following presentation, we go through the different parts of the model, starting at the top of Table 1. The parameter 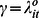, representing infection from other, non-specified sources (Eq. (2)), has to be interpreted relative to infection within and between farms. Infection from other, non-specified sources accounted for only 6% of the lice abundance, as calculated from Eq. (7). Infection from neighbourhood farms accounted for a further 28% of the lice abundance, whereas the remaining 66% was attributed to infection within farms. The confidence intervals for these proportions are all narrower than 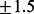 %. Note that the latter includes both within farm infection of infectious copepodite-stage salmon lice and pre-adult and adult stages of lice that survive from previous counts.

All four lags of observed lice abundance (Eq. (4) and Eq. (5)) were selected. Deleting the last three lags would increase the BIC value by 688, so including these lags improves the fit considerably. However, the estimated values of 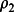, 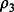 and 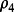 sum up to 0.12, so these lags only account for 11% (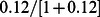) of the weighted observed lice abundance.

The estimated value of 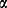 ((Eq. (4) and Eq. (5)) was 0.65, which means that the expected lice abundance was not proportional to lagged observed lice abundance. Doubling the lagged observed lice abundance corresponds to a 57% increase in the expected lice abundance (since 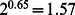).

**Figure 4:**
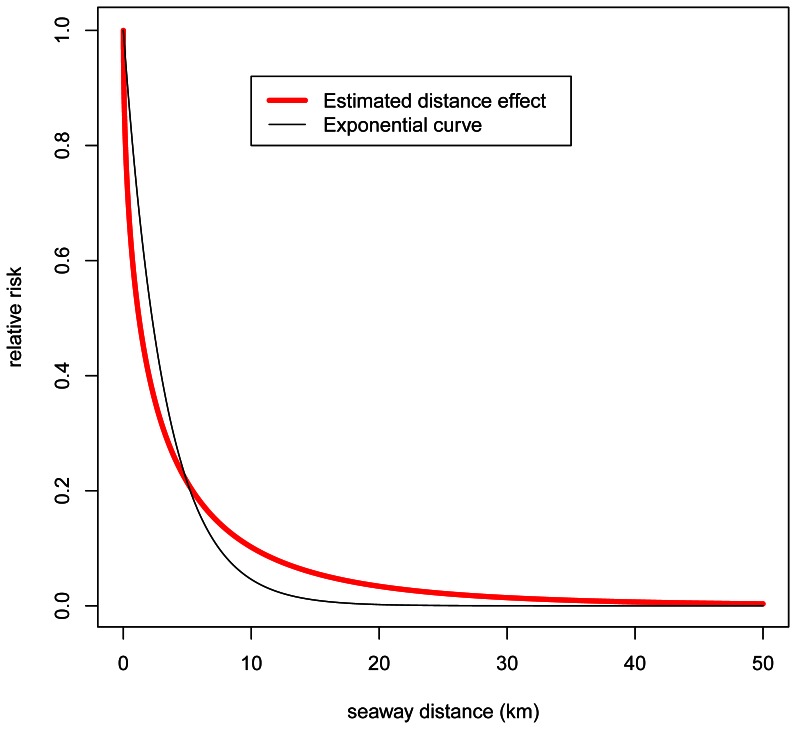
Estimated relative effect of the seaway distance.

The infection pressure from neighbourhood farms decreased sharply by increasing seaway distance (Figure 4). A value equal to 1 for the parameter 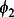, which controls the Box-Cox transformation, would correspond to an exponential distance function, which we assumed in previous work on infectious diseases with less informative data [5], [16]. However, 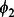 was estimated to be much lower than 1, which gives a much steeper curve than the exponential.

We continue by commenting on the effect of explanatory variables included in the factor 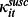 (Eq. (3)). All the variables included here were measured in the current month *t*, except the seawater temperature difference between month *t* and 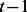. The 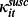 term includes a function of time common to all farms, represented by a third order polynomial. Deleting the time trend (represented by three terms) from the model would increase the BIC value with only 46, so the time trend is not among the most important factors for improving the model fit.

**Figure 5:**
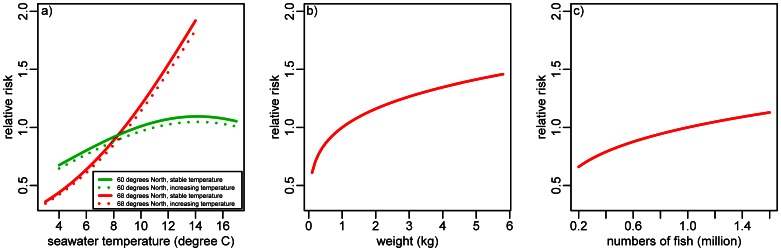
Estimated relative effects of the seawater temperature (a), of the mean fish weight (b) and of the number of fish at neighbouring farms (c).

The factor 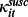 is also a function of several characteristics of the susceptible farm. The most important is the seawater temperature, which is represented by a second order polynomial of temperature, a cross product of temperature and latitude and a difference between the temperatures at month 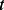 and 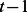. Deleting these temperature terms from the model would increase the BIC value with 2395, implying that water temperature is a major predictor of salmon lice abundance. Figure 5a) shows the estimated combined effects of the seawater temperature and the latitude for farms at 60° North (close to the city of Bergen) and 68° North (in the Lofoten area). The seawater temperature dependency was much stronger in northern Norway than in southern Norway. In addition, in southern Norway, the effect flattened out when the seawater temperature reached about 14°C. There was also a smaller, but statistically significant, effect of the seawater temperature increase from the previous month, which reduced the expected lice abundance slightly. In practice, this means that the effect of the nominal seawater temperature in periods with increasing seawater temperatures (typically spring and summer) differs from that in periods with decreasing seawater temperatures (autumn and winter).

The mean fish weight at susceptible farms is a very important explanatory variable, with a relative BIC value of 967. Since it enters in Eq. (3) as the logarithm of the mean fish weight, it has the form 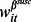 after taking the exponential, where 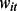 is the mean fish weight and 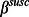 the regression coefficient, which was estimated to be 0.215. Figure 5b) illustrates the effect of the mean fish weight.

When a farm was active in month 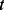, but not in month 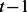, the fish cohort at the farm was considered as stocked if the mean fish weight was less than 250 g and relocated otherwise. This was modelled by including corresponding indicator variables in Eq. (3). Remember that previous lice counts in this case is 0. If a fish cohort was stocked, its expected lice abundance was reduced by 62% (since 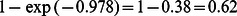) compared to a farm that was active in the previous month, but with 0 observed lice abundance. On the other hand, if a fish cohort was relocated, the comparable expected lice abundance increased by 24% (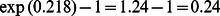). This is probably because relocated fish bring with them infection.

The expected lice abundance for a salmon cohort was 14% (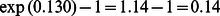) higher than for a cohort of rainbow trout, if they had the same lagged observed lice abundance and everything else being equal.

The number of fish was the only characteristic of the infectious farms that was included in the sub-model for infectiousness (Eq. (6)). The infection pressure from neighbourhood farms increased by increasing numbers of fish at such farms since 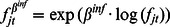, where 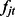 is the number of fish at a neighbouring farm and 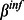 the regression coefficient, which was estimated to be 0.258. Figure 5c) illustrates the effect of the number of fish at neighbouring farms.

We finally report the results for models including medical lice treatment. Extending Eq. (3) with an indicator variable for medical treatment in the current month improved the BIC value with 352. However, the estimated coefficient was 0.28 (95% confidence interval from 0.25 to 0.31), which is the opposite sign of the expected causal relationship. If we instead include an indicator variable for medical lice treatment in the previous month, the estimated coefficient was −0.34 (95% confidence interval from −.37 to −0.31), with an improvement in BIC value of 435. Hence medical lice treatment is potentially an important explanatory variable, but until we get more information on the timing and nature of medical treatments, this effect is not trustworthy. It does, however, make sense to include treatment in the previous month in the present data. The salmon farmers are instructed to report the highest abundance counted during a month and it is likely that this count is obtained before a potential medical treatment in a given month. Nevertheless, there is probably still some bias in the estimated effect of medical treatment. Inclusion or exclusion of medical treatment did not, however, substantially affect estimates of other parameters in the model (Table B in Appendix S1).

## Discussion

The main structure of the present model is similar to models we have applied previously for viral diseases in salmonid farms [5], [15], [16] and is in addition inspired by models applied for other animal diseases [1]–[3], in that the distances to neighbouring farms as well as conditions at these farms are important factors for the infection pressure. However, the stochastic modelling differs, which is natural since the response data are different. The viral infection data are binary, i.e. a farm is considered to be infected or non-infected, whereas the salmon lice infection data consist of counts that may take positive values and that are available for every farm-month. This results in a comparatively informative dataset, which has given us the opportunity to account for many different factors and model some of them in detail. Our model also has similarities to models used for human infections. For instance, Held and Paul [29] modelled the monthly number of laboratory cases of influenza in 140 districts of Southern Germany by a spatio-temporal negative binomial regression model, taking into account infection within and between regions (termed epidemic components) and an additional external infection source (called endemic component).

The model can be seen as a generalisation of vector autoregressive models [30], which are widely used in areas such as econometrics: Let 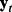 denote the 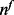-dimensional vector of salmon lice counts for all farms in month 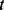, 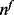 being the number of farms. If the parameter 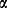 in Eq. (4) and Eq. (5) is exactly 1, the expected lice counts at month 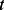 is a weighted sum of the previous lice counts up to 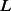 lags back. This can be written as
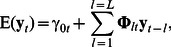
(8)where 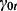 is an 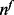-dimensional vector of time-varying farm-specific intercepts representing infection from other, non-specified sources, whereas 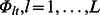 are time-varying 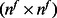 matrices, where the diagonal represents within-farm infection and the off-diagonal elements represent between-farm infection.

The present modelling approach gives insight into the different contributing sources of salmon lice infection, as well as factors that affect lice abundance. Within farm infection was the dominating source of infection, accounting for 2/3 of the additive contribution to salmon lice abundance. The large within farm contribution to infection may partly be due to survival of pre-adult and adult lice over consecutive counts, since these stages are constituents of this internal source in addition to infectious copepodite-stage salmon lice produced within the farm stocks of salmonids. In contrast, the external sources of infection for a given farm constitute only infectious copepodite-stage salmon lice produced externally and transported passively with the water current. It is also important to note that the contribution from the different sources of infection will vary over time and space. As smolts are transferred from freshwater to marine waters, for example, they are free of salmon lice. Transmission of infectious copepodites will then only come from external sources. Hence, externally produced infection, which depends primarily on the density of salmon lice in neighbouring farms in the model, are important for seeding susceptible farm populations of salmonids with infection while the on-farm abundance of reproductive lice is low. The relative contribution from the within farm source of infection increases following the development of reproductive lice, and this source seems mainly responsible for amplifying and sustaining farm populations of salmon lice. The large contribution from within farm infection implies that the average farm primarily depends on itself in avoiding high levels of salmon louse infections. This, we argue, has important implications for the control and management of salmon lice. Due to the importance of self-inflicted infection, the farmer should be motivated to control infection levels of reproductive lice to minimise the production of infectious copepodites. From a management point of view, it is reasonable to hold the farms responsible for keeping levels of reproductive lice low, to minimise their contribution to local infection pressure to wild fish or neighbourhood salmon farms.

The external unknown source of infection accounted for only 6% of the additive contribution to salmon lice abundance. This source may represent infection originating from wild salmonids [9], [31], or alternatively escaped farmed salmonids [32], but which we do not have information on in the present study. The external unknown source is modelled with fewer parameters than the other sources of infection, and it is possible that it would account for a larger proportion of the infection if a more flexible function was used. However, we did not find any logical reasons to do so. The low contribution from the unknown external source also agrees with earlier suggestions that farmed salmon must be the dominating host of salmon lice in Norwegian coastal waters due to the large population size compared to wild stocks [11], [32].

Infection from external neighbourhood farms accounted for the final 28% of the additive contribution to salmon lice abundance. The contribution to a given farm by a neighbourhood farm in the model is shaped by the functional relationship between the relative risk and seaway distance between farms (Figure 4). Compared to the exponential function used in previous models [5], [15], [16], the present relative risk decreases steeply at low but increasing distance to neighbourhood farms, but levelled off at higher relative risks for intermediately distant neighbourhood farms. We interpret this to imply that i) very close farms on average interact intensively with respect to salmon lice infection, and ii) that the number of intermediately distant neighbourhood farms and their levels of infection are important for expected salmon lice abundance. The latter probably reflects the effect of being located in areas of intensive salmon farming [11]. The function describing the relationship between infection from neighbourhood farms and distance represents an expression of a transmission kernel for salmon lice transmission between neighbourhood farms that can be used to simulate the spread of infection between farms and assess the impact of different control measures. Such transmission kernels are key elements of models designed to examine scenarios for the spread and control of a range of different livestock diseases (e.g. [4]). To our knowledge, this is the first transmission kernel estimated for metazoan parasites from an extensive system of farm populations. It is worth noting, however, that the shape of the function describing the transmission kernel for salmon lice probably varies in space and time, for example influenced by the temperature dependent duration of the planktonic phase of the louse life cycle [33], and local hydrodynamics [34].

The large body of data used to parameterise the present model yield estimates with relatively tight confidence intervals. The reports of salmon lice abundance, however, are highly skewed and zero inflated, implying that modelled expected abundance of lice will have relatively large uncertainty while predicting individual lice reports. It is also worth noting that the farmers are instructed to report the highest count during a month such that the modelled expectations should tend to be overestimates of the true lice abundance at corresponding times. In addition, there is one general characteristic of the data that we suspect may be confounded by the regulation regime authorised by the legal authorities in Norway [11]. For the most influential factors in the model, effects seem relatively reduced for factor-values that imply expectations of high salmon lice abundance, i.e. effects are less than proportional to changes in the factors. This is apparent for example at especially high temperatures in the south, for large fish and for neighbourhood farms with large numbers of fish (Figure 5). Bearing in mind that the regulations on salmon lice infections focus on maximum legal thresholds of parasite abundances on farms, we anticipate that control efforts like the use of cleaner fish or medical treatments counter further increases in infection levels as lice abundances exceed legal thresholds. Since we cannot fully account for control efforts with the present resolution of data, this type of confounding related to legal thresholds of infection is to be expected. On top of this, we suspect that there may also be some underreporting of high salmon lice abundance. For example, the probability of finding a given salmon louse on a fish, i.e. the sensitivity of the counting procedure, probably decreases with increasing surface area to search on large fish. An implication of such potentially uncontrolled effects is that the model would be less predictive for high than for low lice abundance. Higher resolution of the data concerning timing and the nature of control measures, along with increased knowledge on the dynamic effects of different measures, are aspects that would improve the predictive capability of the present approach.

To conclude, we present a stochastic spatio-temporal model for salmon lice infection on farmed salmonids in Norway. We emphasise important insights that the model provides with respect to different sources of infection and how salmon lice abundance is affected by different environmental -, host related - and parasite related factors. In addition, we address control measures such as medical treatments and the use of cleaner fish, but point to shortcomings of the data to fully account for control measures at present. The present modelling framework lays the foundation for development of future scenario simulation tools for examining transmission and abundance of salmon lice on farmed salmonids under different control regimes. From 2012, the reporting procedures for salmon lice infections and medical treatments have changed from a monthly to a weekly frequency. With this increased time resolution we foresee that we can estimate more trustworthy effects of medical treatments and other control measures. This will benefit the prospects of applying the present model as a mathematical laboratory to investigate effects of complex and expensive actions before they are implemented in practice.

## Supporting Information

Appendix S1 (PDF)
