# Supplementary material for: Space-Time Modelling of the Spread of Salmon Lice between and within Norwegian Marine Salmon Farms
Source: PLoS One. 2013 May 20;8(5):e64039. doi: 10.1371/journal.pone.0064039 (PMC3659056; doi:10.1371/journal.pone.0064039)
Supplement: Appendix S1 — (PDF) [file pone.0064039.s001.pdf]

Appendix to:
Space-time modelling of the spread of salmon lice between and within Norwegian marine salmon farms

Magne Aldrin[1,2,*], Bård Storvik[1], Anja Bråthen Kristoffersen[3,4] Peder Andreas Jansen[3]

1 Norwegian Computing Center, Oslo, Norway

2 Department of Mathematics, University of Oslo, Oslo, Norway

3 Norwegian Veterinary Institute, Oslo, Norway

4 Department of Informatics, University of Oslo, Oslo, Norway

∗ **E-mail: magne.aldrin@nr.no**

Modelling the excess zero probability

The probability for excess zeroes is modelled by logistic regression. A Box-Cox transformation of the expected lice abundance, μ_{it}, is used as an explanatory variable. This is a way to account for all important variables in a parsimonious way. In addition, we take into account if the farm of current interest was active the previous month and, if so, if it had zero lice counts or not. More formally, the probability for excess zeroes is given by

$$\begin{aligned}\text{logit}(p^z_{it}) &= \log(p^z_{it}/(1-p^z_{it})) \\ &= \beta^z_0 + \beta^z_1 \cdot (\mu_{it}^{\beta^z_2} - 1)/\beta^z_2 + \beta^z_3 \cdot y^0_{i(t-1)} \cdot S_{i(t-1)} + \beta^z_4 \cdot (1 - S_{i(t-1)}).\end{aligned} \quad (1)$$

Here, $y^0_{i(t-1)}$ is an indicator variable that is 1 if $y_{i(t-1)} = 0$, i.e. if there was a zero lice count at farm i at month $t-1$, and 0 elsewhere. Furthermore, the β^z-s are coefficients as usual. The interpretation of the first and the last two terms in (1) is that the sum of these constitute three different intercepts. The intercept is β^z_0 if the current farm was active in the previous month ($S_{i(t-1)} = 1$) with positive lice counts ($y^0_{i(t-1)} = 0$). If zero lice was counted in the previous month ($y^0_{i(t-1)} = 1$), the intercept is $\beta^z_0 + \beta^z_3$. Finally, if the current farm was in-active in the previous month ($S_{i(t-1)} = 0$), the intercept is $\beta^z_0 + \beta^z_4$. The Box-Cox transformation allows for a non-linear dependency of the expected lice abundance μ_{it} on the logit scale, and β^z_2 controls this non-linear dependency.

Modelling the negative binomial distribution

If X is negative binomially distributed with parameters R and P, its probability distribution is [1]

$$P(X = x) = \frac{\Gamma(x + R)}{x! \cdot \Gamma(R)}(1 - P)^R P^x. \tag{2}$$

The mean is $RP/(1 - P)$ and the variance is $RP/(1 - P)^2$. If we introduce the i and t indexes, in our case with a zero-inflated negative binomial distribution the mean in the negative binomial part is $\mu_{it}^{NB} = R_{it}P_{it}/(1 - P_{it}) = n \cdot \mu_{it}/(1 - p_{it}^z)$, where $n = 20$ is the number if fish in the sample, and the expected salmon lice abundance μ_{it} and the excess zero probability p_{it}^z are modelled as described above. In addition, we model the parameter R_{it} as a function of a Box-Cox transformation of μ_{it} as

$$\log(R_{it}) = \beta_0^R + \beta_1^R \cdot (\mu_{it}^{\beta_2^R} - 1)/\beta_2^R, \tag{3}$$

where the β^R-s are coefficients that are to be estimated from the data. A simpler alternative would be to model R_{it} as a constant, i.e. with $\beta_1^R = 0$, but this would give a significantly poorer fit and provide a less precise description of the probability distribution for the counts. On the other hand, R_{it} could also have been modelled as a separate function of the explanatory variables as in Jansen et al. ([2], but our alternative model in Eq. (3) is a parsimonious compromise between these two extremes.

The parameter P_{it} is then implicitly given as

$$P_{it} = \mu_{it}^{NB}/(\mu_{it}^{NB} + R_{it}) = n \cdot \mu_{it}/(n \cdot \mu_{it} + (1 - p_{it}^z) \cdot R_{it}). \tag{4}$$

The likelihood

We drop the farm and month indexes for a moment and let p^0 denote the probability for the number of counted lice, y, being 0. This is the sum of the probability for excess zeroes and of the probability for an "ordinary" zero from the negative binomial distribution, given by

$$p^0 = p^z + (1 - p^z)(1 - P)^R. \tag{5}$$

The probability for the zero-inflated negative binomial distribution is then

$$P(Y = y) = (p^0)^{y^0}[(1 - p^0)\Gamma(y + R)(1 - P)^R P^y/(y!\Gamma(R)]^{(1-y^0)}, \tag{6}$$

where, as before, $y^0 = 1$ if $y = 0$ and $y^0 = 0$ if $y > 0$.

The log likelihood of our data is therefore

$$\begin{aligned} ll(\boldsymbol{\theta}) = \sum_i \sum_t y^0_{it} \log(p^0_{it}) + (1 - y^0_{it})[\log(1 - p^0_{it}) + \log(\Gamma(y_{it} + R_{it})) \\ + R_{it} \log(1 - P_{it}) + y_{it} \log(P_{it}) - \log(\Gamma(R_{it}))], \end{aligned} \tag{7}$$

dropping terms that do not depend on the parameters.

Results for p_{it}^{x} and R_{it}

Table A shows the estimated values for the parameters in the sub-models for p_{it}^{z} and R_{it}. The probability for excess zeroes increased if the observed lice abundance the previous month was 0 ($beta_3^z > 0$) and when the current farm was in-active the previous month $\beta_4^z > 0$. The probability for excess zeroes increased also by increasing expected lice abundance, which at first glance may seen counter-intuitive. But this does not mean that the total probability for observing 0 lice abundance increased, since the probability for "ordinary" zeroes in the negative binomial distribution at the same time increased. The R parameter in the negative binomial distribution decreased by increasing expected lice abundance, since β_1^R was positive when β_2^R was negative.

Table A. Estimated parameters in the sub-models for p_{it}^{z} and R_{it} with 95 % confidence intervals for the selected model.

Variable name or parameter description	Parameter symbol	Est.	Lower	Upper
Excess zero probability	β_0^z	-3.402	-3.582	-3.222
-"-	β_1^z	0.064	0.010	0.117
-"-	β_2^z	0.326	0.091	0.562
-"-	β_3^z	3.010	2.899	3.121
-"-	β_4^z	3.270	3.098	3.442
R parameter	β_0^R	-2.191	-2.340	-2.042
-"-	β_1^R	1.484	1.311	1.657
-"-	β_2^R	-0.634	-0.687	-0.581

Est.: Estimate
Lower: Lower bound of 95 % confidence interval
Lower: Upper bound of 95 % confidence interval

Results including medical treatment the previous month

Table B corresponds to Table 1 in the main text and shows the estimated parameters in the expected abundance when medical treatment the previous month is included in the model.

Table B. Estimated parameters in the expected abundance μ_{it} with 95 % confidence intervals for the selected model extended with medical treatment the previous month.

Parameter group	Variable name or parameter description	Farm specific variable	Parameter symbol	Est.	Lower	Upper
misc.	Other sources		γ	0.074	0.066	0.082
-”-	Lagged lice counts		ρ_2	0.119	0.101	0.138
-”-	-”-		ρ_3	0.029	0.019	0.040
-”-	-”-		ρ_4	0.024	0.015	0.033
-”-	Non-linear dependency		α	0.683	0.668	0.698
-”-	Sea distance function		ϕ_0	-1.462	-1.619	-1.306
-”-	-”-		ϕ_1	-0.353	-0.277	-0.428
-”-	-”-		ϕ_2	0.567	0.481	0.653
susc.	intercept	no	β_k^{susc}	-0.377	-0.430	-0.323
-”-	$(t - 103/2)$	-”-	-”-	$3.41 \cdot 10^{-3}$	$2.47 \cdot 10^{-3}$	$4.36 \cdot 10^{-3}$
-”-	$(t - 103/2)^2$	-”-	-”-	$5.21 \cdot 10^{-5}$	$3.82 \cdot 10^{-5}$	$6.60 \cdot 10^{-5}$
-”-	$(t - 103/2)^3$	-”-	-”-	$-1.75 \cdot 10^{-6}$	$-2.26 \cdot 10^{-6}$	$-1.24 \cdot 10^{-6}$
-”-	(temp − 9)	yes	-”-	0.1025	0.0983	0.1066
-”-	$(\text{temp} - 9)^2$	-”-	-”-	-0.0050	-0.0059	-0.0041
-”-	(latitude-64)	-”-	-”-	0.0099	0.0057	0.0142
-”-	(temp-9)x(latitude-64)	-”-	-”-	0.0129	0.0118	0.0140
-”-	$\text{temp}_t - \text{temp}_{t-1}$	-”-	-”-	-0.0238	-0.0296	-0.0180
-”-	log(weight)	-”-	-”-	0.224	0.210	0.237
-”-	Stocked	-”-	-”-	-1.082	-1.222	-0.942
-”-	Relocated	-”-	-”-	0.188	0.071	0.305
-”-	Salmon proportion	-”-	-”-	0.152	0.106	0.199
-”-	treatment_{t-1}	-”-	-”-	-0.341	-0.372	-0.310
inf.	log(number of fish)	yes	β_k^{inf}	0.252	0.186	0.318

misc.: Miscellaneous parameters
susc.: Parameters related to the susceptible farm
inf. : Parameters related to the infectious farm
Est.: Estimate
Lower: Lower bound of 95 % confidence interval
Lower: Upper bound of 95 % confidence interval

Other, non-optimal variants of the model

We also investigated several variants of the model, but none of these gave improved BIC values compared to the selected model. These include:

- Models with either a third order polynomial of seawater temperature, a cross product of latitude and squared temperature, or a second order polynomial of the logarithm of the fish weight.
- A model with seawater temperatures from month $t-1$ instead of month t.
- A model with the logarithm of the number of fish at the susceptible farm as an explanatory variable in Eq. (3) in the main text.
- A model with the logarithm of (seawater temperature + 0.6) as used by Jansen et al. [2].
- The procedures for reporting lice counts were changed in August 2009, and this could potentially have given lower lice counts. We therefore fitted a model with an indicator variable for August 2009 in Eq. (3) in the main text, and another model with an additional indicator variable for September the same year.
- The lice counts show a strong seasonal variation, which potentially can be accounted for by the seawater temperature. To investigate if there was further seasonal variation, we fitted a model with a pair of sine and cosine functions with a period of 12 months in Eq. (3) in the main text, and a model that in addition included a pair with a period of six months.

References

1. Johnson N, Kotz S, Kemp A (1993) Univariate discrete distributions. John Wiley & Sons, Inc., New York.

2. Jansen P, Kristoffersen A, Viljugrein H, Jimenez D, Aldrin M, et al. (2012) Sea lice as a density dependent constraint to salmonid farming. Proc Biol Sci rspb20120084.
